# Physical Violence during Pregnancy and Its Implications at Birth: Analysis of a Population Survey, 2019

**DOI:** 10.3390/healthcare11010033

**Published:** 2022-12-22

**Authors:** Laura Virginia Periche Medrano, María Alejandra Guerrero Loarte, Fabriccio J. Visconti-Lopez, Diego Azañedo, Rodrigo Vargas-Fernández

**Affiliations:** 1 Facultad de Ciencias de la Salud, Universidad Científica del Sur, Lima 15024, Peru; 2 Facultad de Ciencias de la Salud, Universidad Peruana de Ciencias Aplicadas, Lima 15023, Peru

**Keywords:** low birth weight, abortion, physical violence, pregnancy, Peru

## Abstract

Physical partner violence is widely recognized as a global health problem, especially in pregnant women. This study determines the association between physical violence during pregnancy in Peruvian women aged 15 to 49 years with low birth weight and abortion according to the Demographic and Family Health Survey (ENDES) 2019. An analytical cross-sectional observational study was carried out based on the data from the ENDES 2019. Two dependent variables referring to birth outcomes were included: abortion and low birth weight. The independent variable was physical violence during pregnancy by her current or former husband/partner. A total of 15,305 women were included in the study. The prevalence of physical violence during pregnancy was 6.43%. Regarding the adverse outcomes of pregnancy, the prevalences of abortion and low birth weight were 20.84% and 6.01%, respectively. Women suffering physical violence during pregnancy were more likely to have an abortion but not low birth weight. In conclusion, it was found that 6 in 100 Peruvian women of childbearing age were victims of violence during pregnancy. Likewise, it was observed that women who were victims of violence during pregnancy had a higher probability of having an abortion but not low birth weight.

## 1. Introduction

Physical violence during pregnancy is a serious public health problem that can have serious consequences for the mother and the newborn [1]. According to a meta-analysis published in 2021, the global prevalence of physical violence during pregnancy is estimated at 9.2%. Similarly, the prevalence was highest in Oceania (19.1%) and Africa (16.3%), followed by South America (9.8%) [2]. In Peru, the prevalence of physical violence in pregnant women in 2019 was estimated at 8.6% [3]. In this regard, physical violence during pregnancy could affect the mother indirectly through the development of depression, anxiety, drug abuse, and alcoholism, as well as directly due to possible abdominal damage and placental abruption [1]. All this can place the health of the fetus at risk, causing outcomes such as low birth weight and spontaneous or induced abortion [1]. The high frequency of physical violence during pregnancy requires urgent intervention due to the potential risk of morbidity and mortality for the mother and fetus.

Low birth weight and abortion are highly prevalent problems worldwide. On one hand, according to the United Nations Children’s Fund, in 2015, it was estimated that almost 15% of the 20.5 million births had low birth weight [4]. In this regard, the literature reports that these newborns have an increased risk of mortality during the first year of life and growth retardation, lower intelligence quotient, and an increased risk of chronic diseases in adulthood [5,6,7]. On the other hand, in the period of 2010–2014, it was estimated that at least 35 out of every 1000 women of childbearing age in the world had an induced abortion [8]. In relation to this, unsafe induced abortion is one of the main causes of maternal mortality [9], which is important for women from Latin American countries like Peru, most of whom have little or no access to means of safe abortion because they are not covered by law [10]. Likewise, it is estimated that 1 in 10 women will have a spontaneous abortion throughout their lives, which is equivalent to 23 million spontaneous abortions in the world every year [11]. It is necessary to have evidence of the determinants of this type of event in order to focus on strategies of prevention.

Different studies have reported an association between intimate partner violence and the occurrence of adverse pregnancy outcomes. A study conducted in Tanzania in the years 2001–2002 identified that pregnant women subjected to intimate partner violence were more likely to have a spontaneous abortion (adjusted prevalence ratio (aOR) = 1.60; 95% confidence interval (CI): 1.06–1.60) and more likely to have an induced abortion (aOR = 1.90; 95% CI: 1.30–2.89) [12]. In India, between 2015 and 2016, it was reported that women who had experienced physical partner violence presented a higher risk (adjusted risk ratio (aRR) = 1.5; 95% CI: 1.20–2.00) of having any type of abortion [13]. On the other hand, in a study carried out in Bangladesh in the period 2015–2016, it was reported that exposure to physical violence during pregnancy was associated with having a newborn with low birth weight (aOR = 3.01; 95% CI: 2.35–5.81) [14]. Another study conducted in Tanzania showed the same association (aOR = 3.20; 95% CI: 1.30–7.70) [15]. The evidence on the association between physical violence during pregnancy and this type of outcome is rare in Latin American countries, such as Peru, in which 1 in 10 pregnant women suffers physical violence [3].

Taking this into account, the objective of this study was to evaluate the association between physical violence during pregnancy and abortion and low birth weight in the Peruvian population, using the Demographic and Family Health Survey (ENDES—acronym in Spanish) for the year 2019.

## 2. Materials and Methods

### 2.1. Data Source and Description

A cross-sectional study was carried out with data from the ENDES 2019, which is carried out annually by the National Institute of Statistics and Informatics (INEI—acronym in Spanish). The objective of the ENDES 2019 was to provide information on demographic dynamics, the health status of mothers and children under five years of age residing in the national territory, the fertility of women, non-communicable and communicable diseases, and access to diagnosis and treatment services for the formulation of population and family health and budget programs. This information was collected through three questionnaires: the Household Questionnaire, the Individual Questionnaire, and the Health Questionnaire. The Individual Questionnaire collects information on the sociodemographic characteristics of women aged 12 to 49 years; reproductive history; prenatal care; delivery assistance and puerperal care; pregnancy and lactation; and domestic violence [16]. 

The ENDES 2019 is a nationally representative survey, in which the target population was private households and their members, those who are regular residents, and those who spent the night in the home the night before the day of the survey. In addition, all women aged 15 to 49 years, children under 5 years of age, women aged 12 to 14 years, and people aged 15 years and older in each particular household, as well as all boys and girls under 12 years of age, were included. The sampling framework of the ENDES is made up of the statistical and cartographic information from the National Population Censuses XI and Housing VI for the year 2007 and the Household Targeting System (SISFOH—acronym in Spanish) 2012–2013 Update. Greater details of the methodology can be found in the ENDES report [16].

### 2.2. Sampling and Data Collection

The ENDES 2019 sample has a two-stage, probabilistic approach of a balanced, stratified, and independent type, at the departmental level and by urban and rural areas, which gives representative estimates at the national level, by urban/rural area, and by natural region (Metropolitan Lima, coast, highlands, and jungle and in each of the 24 departments of Peru and the Constitutional Province of Callao). The sampling units in the urban areas were the conglomerate and the private dwellings occupied in an urban area, while in the rural areas they were the rural census area and the private dwellings occupied in a rural area [16]. Data collection was carried out by an interviewer who had previously been trained and supervised during an interview in the selected household. This data was recorded on a personal computer and sent to the INEI headquarters [16]. Finally, all women aged 15 to 49 who had complete information in the module on domestic violence and on the characteristics of pregnancy and childbirth were included.

### 2.3. Variables

#### 2.3.1. Outcome Variables

Two dependent variables that refer to birth outcomes were considered. One of the variables was abortion, constructed through the variable V228, which was self-reported by the woman on whether she had ever had a pregnancy that ended in miscarriage, abortion, or stillbirth. When the woman had an affirmative answer to this question, she was categorized as 1. It should be noted that this variable did not allow us to identify whether it was an induced or spontaneous abortion.

The other dependent variable was low birth weight, constructed from variable M19, which was self-reported by the woman and provided knowledge of the weight (in grams) of the child at birth. According to the World Health Organization, low birth weight is defined as a first recorded weight after birth of less than 2500 g [17]; following this definition, low birth weight was classified as 1, with a birth weight of less than 2500 g.

#### 2.3.2. Independent Variable

The independent variable was physical violence during pregnancy, constructed through the variables D118A and D118J, which was self-reported by the woman on whether she was physically abused (ever) during pregnancy by her current or former husband/partner. This variable was categorized and coded as 1 when the woman was physically abused (ever) during pregnancy by her current or former husband/partner.

#### 2.3.3. Explanatory Variables

These variables were selected in accordance with previous studies carried out on the subject [13,14,18,19]. The variables were: age of the woman (in years), educational level (no level/primary, secondary, or higher), marital status (never married, married/living together, or separated/widowed/divorced), ethnic self-identification (native or non-native), employment status (employed or unemployed), prenatal checkups (eight or more or less than eight), iron intake during pregnancy (yes or no), institutionalized delivery (yes or no), number of children (0, 1–3, 4, or more), birth order (1, 2–3, 4, or more), type of pregnancy (single or multiple), desired pregnancy (yes or no), area of residence (urban or rural), wealth quintile (poorest, poor, middle, richer, or richest), and natural region (coast, highlands, or jungle).

### 2.4. Statistical Analysis

All analyses were performed using the Stata 14 software (StataCorp, College Station, TX, USA). The svy command was used to apply sampling weights and stratifications and ensure that the results have a level of inference at the national and regional levels. A descriptive analysis was performed to report the absolute and relative frequencies of the categorical variables and the mean with standard deviation (SD) for quantitative variables that met the normality criterion. The chi-square test was used to determine the differences between the proportions of the sociodemographic characteristics and the dependent and independent variables, and Student’s t-test was used to compare the means of the age variable in the dependent and independent variables. In the inferential analysis, Poisson log generalized linear regression models were used to determine the association between physical violence during pregnancy and the dependent variables and to estimate crude and adjusted prevalence ratios (PR) for the confounding variables (women’s sociodemographic, pregnancy, and childbirth characteristics) together with their 95% CI. Estimation of the PR using a Poisson regression model has been shown to be correct and robust with different prevalences (low and high) in crude and adjusted models, as opposed to odds ratio calculation [20,21,22]. This methodology for estimating a PR has been used in several cross-sectional studies [23,24,25]. Lastly, an estimate was considered statistically significant when the *p* value was less than 0.05.

### 2.5. Ethical Considerations

The approval of an ethics committee was not required because the ENDES data is anonymous and does not reveal any personally identifiable information. Likewise, the ENDES database is freely accessible and can be downloaded free of charge from the platform. The INEI website can be found at the following address: (http://iinei.inei.gob.pe/microdatos/, accessed on 10 August 2022).

## 3. Results

A total of 15,305 women with a pregnancy in the last five years were included in the study. The mean age of the women was 30.59 years (SD: 6.88). Most of the women had a secondary education (45.20%), were married or cohabiting (86.05%), self-identified as non-native (94.02%), and had a job (57.25%). Further details of the sample are shown in Table 1.

The prevalence of physical violence during pregnancy was 6.43%, and the mean age was 31.32 years (SD: 7.14) among those suffering from violence. In addition, the frequency of physical violence was highest among those who did not have an educational level or had only up to primary school education; were separated, widowed, or divorced; and had a job. Regarding the characteristics of the pregnancy, physical violence frequency was highest in those with fewer than eight prenatal checkups, those with four to seven children, and those who did not want to get pregnant. Women residing in the poorer and middle wealth quintiles had the highest frequencies of physical violence in comparison with other categories of wealth quintile (Table 2).

Regarding birth outcomes, the prevalences of abortion and low birth weight were 20.84% and 6.01%, respectively. In relation to physical violence during pregnancy, we observed that the proportions of abortion and low birth weight in women who were exposed to violence were 30.52% and 5.30%, respectively. The frequency distributions of abortion and low birth weight according to sociodemographic, pregnancy, and household characteristics can be seen in Table 3.

In the crude model, an association was found between physical violence during pregnancy and abortion (PR: 1.51 (95% CI: 1.32–1.73)), while low birth weight did not show a statistically significant association (*p* = 0.420). In the adjusted model, it was found that women who had been physically abused during pregnancy were more likely to have an abortion (adjusted prevalence ratio (aPR): 1.43 (95% CI: 1.24–1.64)), while low birth weight did not show statistical significance (*p* = 0.174) (Table 4).

## 4. Discussion

The study sought to assess the association between physical violence during pregnancy in Peruvian women and adverse birth outcomes. The findings of this study indicate that approximately 6 in 100 women experienced physical violence during pregnancy. Regarding the adverse outcomes of pregnancy, it was found that 1 in 5 pregnancies ended in abortion, and 1 in 20 newborns had low birth weight. Regarding the examination of the association between the variables of interest in the study, women experiencing violence during pregnancy had a higher probability of abortion compared to their counterparts. However, this was not associated with low birth weight.

Physical violence is experienced by 6.43% of Peruvian women. This figure is less than that evidenced in a systematic review that reported an average global prevalence of 9.2% [2]. Likewise, this prevalence is within the ranges reported in the Latin American and Caribbean (LAC) region, which vary from 4.7% (Brazil) to 43.8% (Mexico) [26]. The prevalence of physical violence during pregnancy is estimated to have decreased in the Peruvian territory since a study of hospital reports carried out in 2008 reported that the prevalences of physical violence in women with a planned and unplanned pregnancy were 26.6% and 32.6%, respectively [27]. In addition, some characteristics which may lead Peruvian women to suffer violence have been described, such as living in highlands or jungle, having a secondary education, having children, alcohol consumption by their partners, and a family history of violence [28]. Also, another very important factor is having suffered abuse during childhood; in Peru, high levels of prevalence of child abuse have been described (67.1%), and it has been observed that having experienced physical abuse during childhood increases the probability of experiencing emotional, physical, sexual, and any type of violence in adulthood [29]. This prevalence, which is less than 7%, could be attributed to the fact that the Peruvian State has a regulatory framework and public policies (National Plan Against Gender Violence 2016–2021) that protect the human rights of women and consider the eradication of violence against women a priority, indicating the powers of the central government and regional and local governments to deal with this problem. Among them are: the Political Constitution of Peru; the Law of Equal Opportunities between Women and Men; the General Law of Health; the Protection Law against Family Violence; the Penal Code; the Law for the Prevention and Punishment of Sexual Harassment; and the Law against Human Trafficking and Migrant Smuggling [30]. Likewise, there are Women’s Emergency Centers, one of the few specialized and free public services in Peru, as well as free emergency telephone lines (Line 100), which operate 24 h a day throughout the year in relation to this problem [31]; however, it is not possible to establish whether these measures have had an impact on the decrease in the prevalence of violence against women.

Regarding the prevalence of abortion, it was found that the pregnancies of 20.84% of Peruvian women of childbearing age ended in abortion. This result is lower than that reported in the LAC region (32%), which has the highest prevalence of abortion, followed by the European region (30%), Asia (28%), and North America (17%), which could be attributed to under-reporting by the region or social desirability biases [8]. On the other hand, in this study 6.01% of the participants had a newborn with low birth weight, which is lower than the world average (14.6%) and that of the LAC region (8.7%) [32]. These figures could be explained by the fact that, in recent years, in Peru, new measures have been implemented that may affect certain maternal health factors (such as prenatal check-ups, iron intake, and institutionalized childbirth) and demographic factors (such as the area of residence, wealth quintile, and natural region), which were found to be significant in our study [33]. In particular, to overcome the economic barrier, the Maternal and Child Insurance was implemented, which guaranteed free delivery care in public health establishments. The Maternal and Child Insurance later became the Public Health Insurance [34]. This insurance provides coverage for prenatal check-ups for pregnant women in national territory; and it is a financial protection for members of the population with fewer economic resources, which must be financially sustainable over time to achieve the goal of preserving the health of mothers and children [35]. Also, maternal health programs provide cash transfers to people with high rates of poverty, people living in rural areas, vulnerable people, and pregnant women on the condition that they access certain health services [36]. On the other hand, to overcome the barrier represented by geographical distances to access maternity care services in rural areas, 500 “Maternity Waiting Houses” were implemented [34]. This is a strategy to provide accommodation to pregnant women, facilitating their access to health facilities, mainly for delivery care, and thus contributing to prevent maternal and perinatal death [37]. Finally, to overcome the cultural barrier, the adaptation of care in maternity services to the customs of mothers was promoted, especially in the context of the cultural diversity existing in the country. Another relevant factor that may have influenced these results is that there has been greater promotion of prenatal care in health centers by increasing the demand for maternal health services [38]. Therefore, these results indicate that interventions regarding maternal health in the country are effective compared to the LAC region, making it necessary to continue promoting these types of measures and even develop new national programs to continue to stimulate prenatal care.

Women who experienced violence during pregnancy were found to be more likely to have had an abortion. However, violence was not associated with lower birth weight. On one hand, studies in low- and middle-income countries have also found that violence during pregnancy is associated with abortion [12,39,40,41]. Likewise, a systematic review reported that violence is also associated with other perinatal health consequences such as premature birth, perinatal death, and premature rupture of membranes [42]. Physical violence during the pregnancy period can also cause physical repercussions such as rupture of the uterus, premature abruption of the placenta, and chorioamnionitis, which are risk factors for spontaneous abortion [43]. Furthermore, stress can disrupt the immune system, resulting in early uterine contractions and the release of prostaglandins, which can lead to abortion in affected women [44]. On the other hand, our study did not find a significant association between violence and low birth weight, a finding which differs from some other published articles. This lack of association could be linked to the fact that in the countries of the LAC region, women have greater social support than in other regions such as Asia and Europe. This factor induces women to engage in fewer high-risk behaviors, such as smoking and drug and alcohol abuse [45,46]. Furthermore, it is possible that women who have more family support will receive appropriate prenatal care [47]. Although the association of abortion with intimate partner violence during pregnancy is adequately documented in the literature, the mechanisms by which these outcomes are produced are not fully understood, which is why further studies are necessary to evaluate the etiology of these outcomes.

This study is not without some limitations. On one hand, being a cross-sectional study, it is not possible to determine the causality of the variables. Being based on secondary data, this study is limited to performing its analysis with the variables that were obtained from the survey database, with other factors that could influence these outcomes, such as the presence of social support or substance use, being lacking. On the other hand, the fact of reporting violence can be a personal and controversial issue, and the study participants may have modified their responses to fit social standards, thus giving rise to a possible social desirability bias. Regarding the abortion variable, the ENDES only collects this information in a general manner without identifying the cause of the abortion. Another limitation is a possible memory bias because the questions are based on past events that occurred at specific moments in life. However, the ENDES 2019 is a nationally representative survey that evaluates these outcomes of interest, which preliminarily exposes physical violence in pregnancy and its association with the implications of childbirth in the Peruvian population.

## 5. Conclusions

It was found that 6 in 100 Peruvian women of childbearing age were victims of violence during their pregnancy in 2019. Likewise, it was observed that those people who were victims of violence during pregnancy had a higher probability of having an abortion. Improvements in maternal and neonatal health require the development and improvement of maternity services but must also necessarily address the structural discrimination and violence that women often experience. Pregnancy is a period in which women are in constant contact with health services, which makes it the ideal time to implement recommendations and launch prevention, awareness, and care mechanisms. It is necessary to reinforce new public health policies in order to reduce violence in pregnancy and its impact on women and newborns in order to reduce both maternal and infant mortality during childbirth.

## Figures and Tables

**Table 1:** Characteristics of the population of women aged 15 to 49 registered in the ENDES 2019.

Characteristic	Population Included in the Study	
	Absolute Frequency (*n* = 15,305)	Weighted Proportion *
Age (in years)		
Mean (SD)		30.59 (6.88)
Education level		
No level/primary	2840	18.05 (17.26–18.86)
Secondary	7124	45.20 (44.13–46.28)
Higher	5341	36.75 (35.63–37.88)
Marital status		
Never married	664	4.26 (3.87–4.69)
Married/living together	13,091	86.05 (85.31–86.76)
Separated/widowed/divorced	1550	9.69 (9.11–10.31)
Ethnic self-identification		
Native	1247	5.98 (5.45–6.55)
Non-native	14,058	94.02 (93.45–94.55)
Employment status		
Employee	8897	57.25 (56.17–58.32)
Unemployed	6408	42.75 (41.67–43.83)
Prenatal checkups		
Eight or more	11,445	74.82 (73.87–75.76)
Less than eight	3860	25.18 (24.24–26.13)
Iron intake during pregnancy		
Yes	14,368	94.58 (94.08–95.03)
No	937	5.42 (4.97–5.92)
Institutionalized childbirth		
Yes	14,560	94.24 (93.61–94.82)
No	745	5.76 (5.18–6.39)
Number of children		
0	4305	28.76 (27.81–29.73)
1–3	10,723	69.55 (68.57-70.51)
4 or more	277	1.69 (1.46-1.96)
Birth order		
1	4823	32.38 (31.45-33.33)
2–3	8067	52.92 (51.92–53.93)
4 or more	2415	14.70 (14.01–15.41)
Type of pregnancy		
Single	15,162	99.14 (98.94–99.30)
Multiple	143	0.86 (0.70–1.06)
Desired pregnancy		
Yes	7336	48.43 (47.39–49.46)
No	7969	51.57 (50.54–52.61)
Area of residence		
Urban	11,006	74.76 (73.84–75.65)
Rural	4299	25.24 (24.35–26.16)
Wealth quintile		
Poorest	3983	22.80 (21.91–23.71)
Poorer	4332	25.49 (24.48–26.53)
Middle	3102	19.92 (19.05–20.82)
Richer	2274	17.08 (16.19–18.00)
Richest	1614	14.71 (1386–15.60)
Natural region		
Coast	6494	55.48 (54.32–56.63)
Highlands	5153	28.02 (26.78–29.30)
Jungle	3658	16.50 (15.53–17.52)

SD: standard deviation. * The weighting factor and the complex sampling of ENDES 2019 were included. SD: standard deviation.

**Table 2:** Characteristics of women aged 15 to 49 years according to whether they experienced physical violence during pregnancy, ENDES 2019.

Characteristic	Physical Violence during Pregnancy		*p*-Value *
	No (*n* = 14,251)	Yes (*n* = 1054)	
Total	93.57 (93.01–94.09)	6.43 (5.91–6.99)	
Age (in years)			
Mean (SD)	30.54 (6.86)	31.32 (7.14)	0.008
Education level			
No level/primary	91.42 (90.11–92.57)	8.58 (7.43–9.89)	<0.001
Secondary	92.60 (91.77–93.35)	7.40 (6.65–8.23)	
Higher	95.83 (94.86–96.62)	4.17 (3.38–5.14)	
Marital status			
Never married	99.84 (99.38–99.96)	0.16 (0.04–0.62)	<0.001
Married/living together	94.38 (93.79–94.91)	5.62 (5.09–6.21)	
Separated/widowed/divorced	83.67 (81.26–85.82)	16.33 (14.18–18.74)	
Ethnic self-identification			
Native	93.16 (91.41–94.57)	6.84 (5.43–8.59)	0.598
Non-native	93.60 (93.01–94.14)	6.40 (5.86–6.99)	
Employment status			
Employee	92.92 (92.18–93.60)	7.08 (6.40–7.82)	0.002
Unemployed	94.44 (93.66–95.13)	5.56 (4.87–6.34)	
Prenatal checkups			
Eight or more	94.06 (93.41–94.65)	5.94 (5.35–6.59)	<0.001
Less than eight	92.12 (90.99–93.11)	7.88 (6.89–9.01)	
Iron intake during pregnancy			
Yes	93.60 (93.02–94.13)	6.40 (5.87–6.98)	0.609
No	93.10 (90.90–94.80)	6.90 (5.20–9.10)	
Institutionalized childbirth			
Yes	93.60 (93.01–94.14)	6.40 (5.86–6.99)	0.691
No	93.19 (90.93–94.91)	6.81 (5.09–9.07)	
Number of children			
0	94.54 (93.47–95.44)	5.46 (4.56–6.53)	0.007
1–3	93.28 (92.63–93.88)	6.72 (6.12–7.37)	
4–7	89.04 (83.54–92.85)	10.96 (7.15–16.46)	
Birth order			
1	96.93 (96.28–97.46)	3.07 (2.54–3.72)	<0.001
2–3	93.05 (92.29–93.74)	6.95 (6.26–7.71)	
4 or more	88.06 (85.95–89.89)	11.94 (10.11–14.05)	
Type of pregnancy			
Single	93.60 (93.04–94.12)	6.40 (5.88–6.96)	0.196
Multiple	90.28 (82.30–94.89)	9.72 (5.11–17.70)	
Desired pregnancy			
Yes	94.78 (94.05–95.43)	5.22 (4.57–5.95)	<0.001
No	92.44 (91.60–93.19)	7.56 (6.81–8.40)	
Area of residence			
Urban	93.69 (93.00–94.31)	6.31 (5.69–7.00)	0.427
Rural	93.23 (92.25–94.10)	6.77 (5.90–7.75)	
Wealth quintile			
Poorest	92.87 (91.83–93.78)	7.13 (6.22–8.17)	<0.001
Poorer	91.39 (89.96–92.63)	8.61 (7.37–10.04)	
Middle	94.07 (92.96–95.01)	5.93 (4.99–7.04)	
Richer	94.51 (93.05–95.68)	5.49 (4.32–6.95)	
Richest	96.69 (95.55–97.54)	3.31 (2.46–4.45)	
Natural region			
Coast	76.35 (74.94–77.70)	23.65 (22.30–25.06)	<0.001
Highlands	84.55 (83.35–85.68)	15.45 (14.32–16.65)	
Jungle	79.47 (77.76–81.08)	20.53 (18.92–22.24)	

SD: standard deviation. * The chi-square test was used, except for the age variable, where Student’s *t*-test was used. The weighting factor and the complex sampling of ENDES 2019 were included.

**Table 3:** Characteristics of women between 15 and 49 years old according to newborn outcomes, ENDES 2019.

Characteristic	Abortion		*p*-Value *	Low Birth Weight		*p*-Value *
	No (*n* = 12,656)	Yes (*n* = 3077)		No (*n* = 14,816)	Yes (*n* = 917)	
Total	79.40 (78.53–80.25)	20.84 (19.75–21.47)		93.99 (93.69–94.64)	6.01 (5.36–6.31)	
Age (in years)						
mean (SD)	30.05 (6.88)	32.63 (6.53)	<0.001	30.58 (6.85)	30.69 (7.35)	0.719
Education level						
No level/primary	80.75 (78.89–82.47)	19.25 (17.53–21.11)	<0.001	91.50 (90.13–92.69)	8.50 (7.31–9.87)	<0.001
Secondary	80.58 (79.38–81.73)	19.42 (18.27–20.62)		94.18 (93.42–94.86)	5.82 (5.14–6.58)	
Higher	76.64 (74.97–78.22)	23.36 (21.78–25.03)		94.97 (94.13–95.70)	5.03 (4.30–5.87)	
Marital status						
Never married	89.51 (85.73–92.38)	10.49 (7.62–14.27)	<0.001	92.08 (88.90–94.40)	7.92 (5.60–11.10)	0.190
Married/living together	78.93 (77.96–79.86)	21.07 (20.14–22.04)		94.13 (93.58–94.64)	5.87 (5.36–6.42)	
Separated/widowed/divorced	76.71 (73.89–79.31)	23.29 (20.69–26.11)		93.56 (91.94–94.88)	6.44 (5.12–8.06)	
Ethnic self-identification						
Native	88.66 (86.13–90.78)	11.34 (9.22–13.87)	<0.001	92.81 (91.13–94.19)	7.19 (5.81–8.87)	0.101
Non-native	78.56 (77.62–79.46)	21.44 (20.54–22.38)		94.07 (93.53–94.56)	5.93 (5.44–6.47)	
Employment status						
Employee	77.86 (76.67–79.01)	22.14 (20.99–23.33)	<0.001	94.39 (93.74–94.98)	5.61 (5.02–6.26)	0.078
Unemployed	80.90 (79.62–82.12)	19.10 (17.88–20.38)		93.45 (32.56–94.24)	6.55 (5.76–7.44)	
Prenatal checkups						
Eight or more	78.35 (77.29–79.37)	21.65 (20.63–22.71)	0.003	95.82 (95.30–96.28)	4.18 (3.72–4.70)	<0.001
Less than eight	81.58 (79.80–83.23)	18.42 (16.77–20.20)		88.56 (87.15–89.82)	11.44 (10.18–12.85)	
Iron intake during pregnancy						
Yes	78.94 (78.01–79.84)	21.06 (20.16–21.99)	0.026	94.15 (93.62–94.64)	5.85 (5.36–6.38)	0.003
No	83.05 (79.59–86.03)	16.95 (13.97–20.41)		91.20 (88.71–93.19)	8.80 (6.81–11.29)	
Institutionalized childbirth						
Yes	78.84 (77.92–79.74)	21.16 (20.26–22.08)	0.005	94.22 (93.70–94.70)	5.78 (5.30–6.30)	0.001
No	84.36 (80.74–87.40)	15.64 (12.60–19.26)		90.24 (86.98–92.75)	9.76 (7.25–13.02)	
Number of children						
0	80.13 (78.40–81.75)	19.87 (18.25–21.60)	0.142	93.49 (92.47–94.39)	6.51 (5.61–7.53)	0.391
1–3	78.88 (77.81–79.92)	21.12 (20.08–22.19)		94.21 (93.59–94.77)	5.79 (5.23–6.41)	
4–7	74.25 (67.34–80.13)	25.75 (19.87–32.66)		93.45 (88.87–96.23)	6.55 (3.77–11.13)	
Birth order						
1	83.87 (82.41–85.23)	16.13 (14.77–17.59)	<0.001	94.34 (93.43–95.13)	5.66 (4.87–6.57)	0.024
2–3	77.77 (76.51–78.97)	22.23 (21.03–23.49)		94.23 (93.50–94.88)	5.77 (5.12–6.50)	
4 or more	73.81 (71.37–76.11)	26.19 (23.89–28.63)		92.37 (90.95–93.58)	7.63 (6.42–9.05)	
Type of pregnancy						
Single	79.15 (78.26–80.02)	20.85 (19.98–21.74)	0.788	94.44 (93.94–94.89)	5.56 (5.11–6.06)	<0.001
Multiple	80.43 (69.72–88.01)	19.57 (11.99–30.28)		42.61 (33.28–52.51)	57.39 (47.49–66.72)	
Desired pregnancy						
Yes	77.31 (75.96–78.61)	22.69 (21.39–24.04)	<0.001	94.06 (93.31–94.72)	5.94 (5.28–6.69)	0.794
No	80.90 (79.72–82.02)	19.10 (17.98–20.28)		93.93 (93.20–94.58)	6.07 (5.42–6.80)	
Area of residence						
Urban	77.13 (76.03–78.20)	22.87 (21.80–23.97)	<0.001	94.62 (94.04–95.14)	5.38 (4.86–5.96)	<0.001
Rural	85.16 (83.84–86.40)	14.84 (13.60–16.16)		92.14 (91.00–93.14)	7.86 (6.86–9.00)	
Wealth quintile						
Poorest	86.53 (85.21–87.75)	13.47 (12.25–14.79)	<0.001	91.91 (90.76–92.92)	8.09 (7.08–9.24)	0.001
Poorer	78.95 (77.17–80.63)	21.05 (19.37–22.83)		94.56 (93.69–95.31)	5.44 (4.69–6.31)	
Middle	79.08 (77.22–80.83)	20.92 (19.17–22.78)		94.67 (93.55–95.61)	5.33 (4.39–6.45)	
Richer	73.52 (71.04–75.87)	26.48 (24.13–28.96)		94.51 (92.96–95.73)	5.49 (4.27–7.04)	
Richest	74.77 (71.91–77.43)	25.23 (22.57–28.09)		94.71 (93.07–95.99)	5.29 (4.01–6.93)	
Natural region						
Coast	76.35 (74.94–77.70)	23.65 (22.30–25.06)	<0.001	94.72 (93.97–95.37)	5.28 (4.63–6.03)	0.002
Highlands	84.55 (83.35–85.68)	15.45 (14.32–16.65)		92.83 (91.89–93.67)	7.17 (6.33–8.11)	
Jungle	79.47 (77.76–81.08)	20.53 (18.92–22.24)		93.52 (92.44–94.46)	6.48 (5.54–7.56)	
Physical violence during pregnancy						
Yes	69.48 (65.43–73.25)	30.52 (26.75–34.57)	<0.001	94.70 (92.78–96.12)	5.30 (3.88–7.22)	0.418
No	79.83 (78.93–80.70)	20.17 (19.30–21.07)		93.94 (93.41–94.44)	6.06 (5.56–6.59)	

SD: standard deviation. * The chi-square test was used, except for the age variable, where Student’s *t*-test was used. The weighting factor and the complex sampling of ENDES 2019 were included.

**Table 4:** Association between physical violence during pregnancy and birth outcomes, ENDES 2019.

Characteristic	Abortion				Low Birth Weight			
	Crude Model		Adjusted Model *		Crude Model		Adjusted Model **	
	PR (95% CI)	*p*-Value	aPR (95% CI)	*p*-Value	PR (95% CI)	*p*-Value	aPR (95% CI)	*p*-Value
Physical violence during pregnancy								
No	Ref.		Ref.		Ref.		Ref.	
Yes	1.51 (1.32–1.73)	<0.001	1.43 (1.24–1.64)	<0.001	0.88 (0.63–1.21)	0.420	0.80 (0.58–1.10)	0.174

PR: prevalence ratio; aPR: adjusted prevalence ratio; CI: confidence interval. * Adjusted for covariates: age, educational level, marital status, ethnic self-identification, employment status, birth order, desired pregnancy, prenatal checkups, iron intake during pregnancy, institutionalized childbirth, area of residence, wealth quintile, and natural region. ** Adjusted for covariates: educational level, prenatal checkups, iron intake during pregnancy, institutionalized delivery, birth order, type of pregnancy, area of residence, wealth quintile, and natural region.

## Data Availability

Not applicable.
